# Evaluating disease activity scores and CBC-derived indices in relation to osteoporosis risk in rheumatoid arthritis: a cross-sectional analysis

**DOI:** 10.3389/fimmu.2026.1774189

**Published:** 2026-08-26

**Authors:** Di Jin, Lina Zhang, Yuwei Wang, Yufeng Pan, Weiduo Nie, Sheng-Guang Li, Yuhua Yan, Ming Li

**Affiliations:** 1 Department of Rheumatology, Weifang People’s Hospital, Weifang, Shandong, China; 2 Department of Rheumatology and Immunology, Peking University International Hospital, Beijing, China; 3 Department of Emergency Internal Medicine, Weifang People’s Hospital, Weifang, Shandong, China; 4 School of Clinical Medicine, Shandong Second Medical University, Weifang, Shandong, China; 5 Dongfang Hospital, Beijing University of Chinese Medicine, Beijing, China

**Keywords:** CBC-derived indices, disease activity, osteoporosis, rheumatoid arthritis, systemic inflammation

## Abstract

**Background:**

Osteoporosis (OP) is a frequent comorbidity in rheumatoid arthritis (RA). This study compared the performance of RA disease-activity scores with complete blood count (CBC)-derived indices for identifying prevalent OP.

**Methods:**

We retrospectively included 226 consecutive patients with RA who underwent dual-energy X-ray absorptiometry (DXA). Predictors comprised age, sex, body mass index (BMI), disease activity (DAS28-ESR, DAS28-CRP, CDAI, and SDAI), ESR, CRP, and six CBC-derived indices (NLR, PLR, MLR, SII, SIRI, and AISI). Logistic and linear regression, receiver-operating-characteristic (ROC) analysis with paired DeLong tests, decision curve analysis (DCA), and four-knot restricted cubic spline (RCS) analyses were performed.

**Results:**

Older age, female sex, lower BMI, and higher CDAI were associated with OP. The five-covariate clinical reference model had an AUC of 0.833. Adding CDAI increased the AUC to 0.892 (ΔAUC, 0.059; P = 0.005), and adding SDAI increased it to 0.877 (ΔAUC, 0.044; P = 0.018), although the ROC curves overlapped substantially. In parallel fully adjusted models, none of the six CBC-derived indices was independently associated with OP (all P≥0.142; apparent AUCs, 0.892–0.895). Adding CRP after DAS28-ESR changed the AUC from 0.863 to 0.869 (ΔAUC, 0.006; P = 0.491). In the extended discrimination model, adding any CBC-derived index changed the AUC by no more than 0.0014 (all P≥0.467). DCA showed small, threshold-dependent differences in net benefit rather than consistent separation between models. Four-knot RCS analyses showed evidence of nonlinearity for DAS28-ESR (P for nonlinearity=0.007) and SDAI (P for nonlinearity<0.001), but not for ln(AISI) (P for nonlinearity=0.391) or ln(SIRI) (P for nonlinearity=0.718).

**Conclusions:**

Disease-activity scores provided modest incremental discrimination beyond established clinical factors, whereas CBC-derived indices added little once conventional inflammatory and disease-activity information was included. These data do not support calculating CBC-derived indices solely to identify prevalent OP or using them as substitutes for guideline-based clinical risk assessment, FRAX, or DXA. The findings do not establish a new screening or treatment threshold.

## Introduction

Rheumatoid arthritis (RA) is a chronic autoimmune disease characterized by persistent systemic inflammation leading to joint destruction and extra-articular complications. Osteoporosis (OP) represents one of its most clinically relevant comorbidities, with a prevalence nearly twice that of the general population (1). Consequently, patients with RA exhibit a substantially higher risk of fragility fractures, particularly vertebral and hip fractures, contributing to morbidity and reduced quality of life (2).

Chronic inflammation is an important contributor to bone loss in RA. Pro-inflammatory cytokines such as TNF-α, IL-1, and IL-6 activate osteoclastogenesis through the RANK/RANKL/OPG pathway and suppress osteoblast function through Wnt/DKK1 signaling (2). Moreover, autoantibodies such as rheumatoid factor and anti-citrullinated protein antibodies can directly promote bone resorption and may precede clinical joint damage (3). These mechanisms collectively result in an imbalance of bone remodeling that may accelerate generalized bone loss even in early or well-controlled RA.

Given the inflammatory basis of RA-associated bone fragility, recent research has explored accessible hematologic biomarkers to quantify systemic inflammation. CBC-derived indices, including the neutrophil-to-lymphocyte ratio (NLR), platelet-to-lymphocyte ratio (PLR), systemic immune-inflammation index (SII), systemic inflammation response index (SIRI), and aggregate index of systemic inflammation (AISI), have emerged as surrogate measures of immune activation (4). These indices integrate innate and adaptive immune-cell components and have been linked to disease activity, cardiovascular outcomes, and mortality in RA (5). Several studies have also shown that elevated NLR or PLR is associated with lower bone mineral density (BMD) and a higher risk of vertebral fracture in postmenopausal women with RA (6), suggesting potential value for identifying bone loss.

In parallel, disease-activity scores—such as the Disease Activity Score in 28 joints (DAS28), Clinical Disease Activity Index (CDAI), and Simplified Disease Activity Index (SDAI)—are validated measures that capture several dimensions of inflammatory burden. Higher disease activity has been associated with reduced BMD and increased fracture incidence, whereas early and sustained disease control may mitigate skeletal deterioration (7, 8). Composite disease-activity scores may therefore reflect the cumulative inflammatory effects relevant to bone metabolism more comprehensively than isolated hematologic indices.

Few studies have directly compared the performance of disease-activity scores and CBC-derived indices for OP in RA. Understanding the magnitude of the additional information provided by each measure may improve interpretation of OP risk assessments. This study therefore performed a head-to-head evaluation of disease-activity scores and CBC-derived indices for identifying prevalent OP among patients with RA. We hypothesized that disease-activity scores would provide greater incremental discrimination than isolated hematologic indices, while recognizing that statistically detectable differences might not be clinically large.

## Methods

### Study design and patients

This was a retrospective, single-center study of patients with RA. We included consecutive patients who fulfilled the 1987 American College of Rheumatology criteria or the 2010 American College of Rheumatology/European League Against Rheumatism classification criteria for RA (9, 10). Patients were excluded if they lacked complete clinical data or had no BMD assessment.

All patients underwent DXA of the radius, lumbar spine (L1–L4), total hip, or femoral neck according to clinical availability. OP was defined as a T-score of −2.5 or lower at any measured diagnostic site, in accordance with World Health Organization criteria (11). For the secondary continuous analysis, the hip T-score was selected because the analytic source was constructed from hip-specific DXA data and provided a common anatomical site across the included patients. This site-selection rule was applied consistently in the linear regression analysis.

### Clinical data collection

We obtained clinical data from medical records, including age, sex, BMI, RA disease duration, menopausal status in female patients, comorbidities, serologic status, and treatment. Comorbid conditions of interest included metabolic or cardiovascular diseases, and the presence of at least one such comorbidity was recorded for each patient. Disease activity was assessed at the time of BMD measurement using DAS28 with ESR (DAS28-ESR), DAS28 with CRP (DAS28-CRP), CDAI, and SDAI. Routine inflammatory markers included ESR (mm/h) and CRP (mg/L). DAS28-CRP was calculated as 0.56×√(tender-joint count) + 0.28×√(swollen-joint count) + 0.36×ln(CRP + 1) + 0.014×patient global assessment + 0.96. CDAI was calculated directly from tender- and swollen-joint counts and the patient and evaluator global assessments; SDAI was calculated as CDAI plus CRP expressed in mg/dL.

A CBC was obtained to determine absolute neutrophil, lymphocyte, monocyte, and platelet counts. Six CBC-derived indices were calculated: NLR = neutrophil count/lymphocyte count; PLR = platelet count/lymphocyte count; MLR = monocyte count/lymphocyte count; SII = platelet count × neutrophil count/lymphocyte count; SIRI = neutrophil count × monocyte count/lymphocyte count; and AISI = neutrophil count × platelet count × monocyte count/lymphocyte count (12–14).

BMD values (g/cm²) and corresponding T-scores were recorded. Ten-year fracture probabilities were obtained using the FRAX tool based on patient risk factors and BMD. The 10-year probabilities of a major osteoporotic fracture and a hip fracture were calculated using the country-specific FRAX algorithm with femoral-neck BMD (15). FRAX measures were used descriptively and were not included in the primary regression models.

### Statistical analysis

Statistical analyses were conducted using Python and R (version 4.2.0). Baseline characteristics stratified by OP status were summarized as mean ± standard deviation or median (interquartile range) for continuous variables and as frequency (percentage) for categorical variables. Group comparisons used Student’s t test, the Mann–Whitney U test, the chi-square test, or Fisher’s exact test, as appropriate.

Predictors associated with OP were evaluated using univariable and multivariable logistic regression, with odds ratios (ORs) and 95% confidence intervals (CIs). Linear regression assessed associations with hip T-score as a continuous outcome. Continuous predictors in **Tables 1** and **2** were standardized to one sample standard deviation, whereas binary predictors retained their original coding.

**Table 1:** Multivariable logistic regression for factors associated with osteoporosis.

Model	Variable	OR	95% CI	P
Model 1	Age (per 1 SD)	2.85	1.96–4.15	<0.001
	Female sex	5.33	1.72–16.51	0.004
	BMI (per 1 SD)	0.39	0.27–0.56	<0.001
	Current glucocorticoid use	1.58	0.80–3.12	0.187
	RF/anti-CCP seropositive	2.31	1.10–4.87	0.027
Model 2	Age (per 1 SD)	2.57	1.69–3.90	<0.001
	Female sex	4.61	1.29–16.49	0.019
	BMI (per 1 SD)	0.49	0.33–0.73	<0.001
	Current glucocorticoid use	0.04	0.01–0.21	<0.001
	RF/anti-CCP seropositive	1.94	0.86–4.39	0.111
	CDAI (per 1 SD)	10.17	3.99–25.93	<0.001
Model 3	Age (per 1 SD)	2.48	1.62–3.78	<0.001
	Female sex	6.53	1.65–25.89	0.008
	BMI (per 1 SD)	0.48	0.32–0.72	<0.001
	Current glucocorticoid use	0.03	0.01–0.18	<0.001
	RF/anti-CCP seropositive	1.67	0.72–3.88	0.237
	CDAI (per 1 SD)	10.77	4.21–27.55	<0.001
	SIRI (per 1 SD)	1.34	0.90–2.00	0.153

Model 1: age, sex, BMI, current glucocorticoid use, and RF/anti-CCP seropositivity. Model 2: Model 1 plus CDAI. Model 3: Model 2 plus SIRI, selected data-informatively for the exploratory model sequence. Continuous predictors are expressed per 1-SD increase. ORs are reported with 95% confidence intervals. Current glucocorticoid use and CDAI were strongly correlated (r=0.839; VIFs, 3.95 and 3.94 in Model 2); the coefficient reversal therefore indicates conditional instability and must not be interpreted as a protective glucocorticoid effect. Parallel CBC-index models and the glucocorticoid-omission sensitivity analysis are reported in **Supplementary Tables 1** and **S2**. BMI, body mass index; CDAI, Clinical Disease Activity Index; CI, confidence interval; OR, odds ratio; SIRI, systemic inflammation response index.

**Table 2:** Multivariable linear regression with hip T-score as a continuous outcome.

Model	Variable	β	95% CI	P
Model 1	Age (per 1 SD)	-0.67	-0.86 to -0.48	<0.001
	Female sex	-1.27	-1.93 to -0.61	<0.001
	BMI (per 1 SD)	+0.68	+0.49 to +0.87	<0.001
	Current glucocorticoid use	-0.44	-0.84 to -0.03	0.034
	RF/anti-CCP seropositive	-0.35	-0.78 to +0.09	0.115
	Adjusted R²	0.352	—	—
Model 2	Age (per 1 SD)	-0.54	-0.72 to -0.35	<0.001
	Female sex	-1.04	-1.67 to -0.40	0.001
	BMI (per 1 SD)	+0.53	+0.34 to +0.71	<0.001
	Current glucocorticoid use	+1.16	+0.45 to +1.87	0.002
	RF/anti-CCP seropositive	-0.22	-0.63 to +0.20	0.302
	CDAI (per 1 SD)	-0.94	-1.30 to -0.59	<0.001
	Adjusted R²	0.421	—	—
Model 3	Age (per 1 SD)	-0.52	-0.71 to -0.33	<0.001
	Female sex	-1.13	-1.78 to -0.49	<0.001
	BMI (per 1 SD)	+0.52	+0.33 to +0.71	<0.001
	Current glucocorticoid use	+1.20	+0.49 to +1.91	0.001
	RF/anti-CCP seropositive	-0.14	-0.56 to +0.28	0.510
	CDAI (per 1 SD)	-0.95	-1.30 to -0.59	<0.001
	SIRI (per 1 SD)	-0.14	-0.33 to +0.05	0.137
	Adjusted R²	0.424	—	—

Model 1: age, sex, BMI, glucocorticoid use, and RF/anti-CCP seropositivity. Model 2: Model 1 plus CDAI. Model 3: Model 2 plus SIRI. Continuous predictors are expressed per 1-SD increase. β coefficients are reported with 95% confidence intervals. β values are expressed in hip T-score units; adjusted R² is shown where supplied. BMI, body mass index; CDAI, Clinical Disease Activity Index; CI, confidence interval; SIRI, systemic inflammation response index.

The regression models were specified hierarchically and were not selected as the best three of all possible variable combinations. Model 1 included age, sex, BMI, current glucocorticoid use, and RF/anti-CCP seropositivity. These covariates were selected on clinical grounds to represent established demographic, body-composition, treatment, and RA-specific factors. Model 2 added CDAI to evaluate clinical disease activity without incorporating an acute-phase reactant. Model 3 added SIRI because it showed the strongest univariable signal among the evaluated CBC indices; this choice was data-informed, exploratory, and not prespecified, and it does not imply that SIRI is superior to the other indices. Correlated disease-activity scores were not entered simultaneously, and no automated all-subsets or stepwise model search was performed.

*Post hoc* model diagnostics evaluated convergence, events per predictor parameter, variance inflation factors (VIFs), and the condition number. Because current glucocorticoid use and CDAI were strongly correlated, a sensitivity model omitted current glucocorticoid use to assess coefficient stability. To avoid relying on the data-informed choice of SIRI alone, six parallel exploratory models separately added standardized NLR, PLR, MLR, SII, SIRI, or AISI to the Model 2 covariate set. The indices were evaluated one at a time because they share blood-cell components; these analyses were not used for automated variable selection (**Supplementary Tables 1**, **S2**).

ROC curves were generated to assess discrimination of prevalent OP. AUC values were compared using paired DeLong tests. For the first comparison, the clinical reference model comprised the same five covariates used in Model 1: age, sex, BMI, glucocorticoid use, and RF/anti-CCP seropositivity. ESR, CRP, and each disease-activity score were then added separately. For the second comparison, the extended clinical/disease-activity reference model comprised these five covariates plus CRP and DAS28-ESR; each CBC-derived index was then added separately. DAS28-ESR was used in the extended reference because it is a widely used composite score that includes joint counts, patient global assessment, and ESR, while CRP was retained as a separate conventional inflammatory marker. SDAI was not used for this purpose because it contains CRP by definition. The extended reference was used to examine the incremental contribution of CBC-derived indices and was not selected on the basis of NRI.

To quantify the additive value of CRP directly, a targeted nested comparison was made between the five-covariate clinical reference plus DAS28-ESR and the same model with CRP added. The AUC difference, 95% CI, and paired DeLong P value were estimated from the same analytic sample.

**Figure 1:**
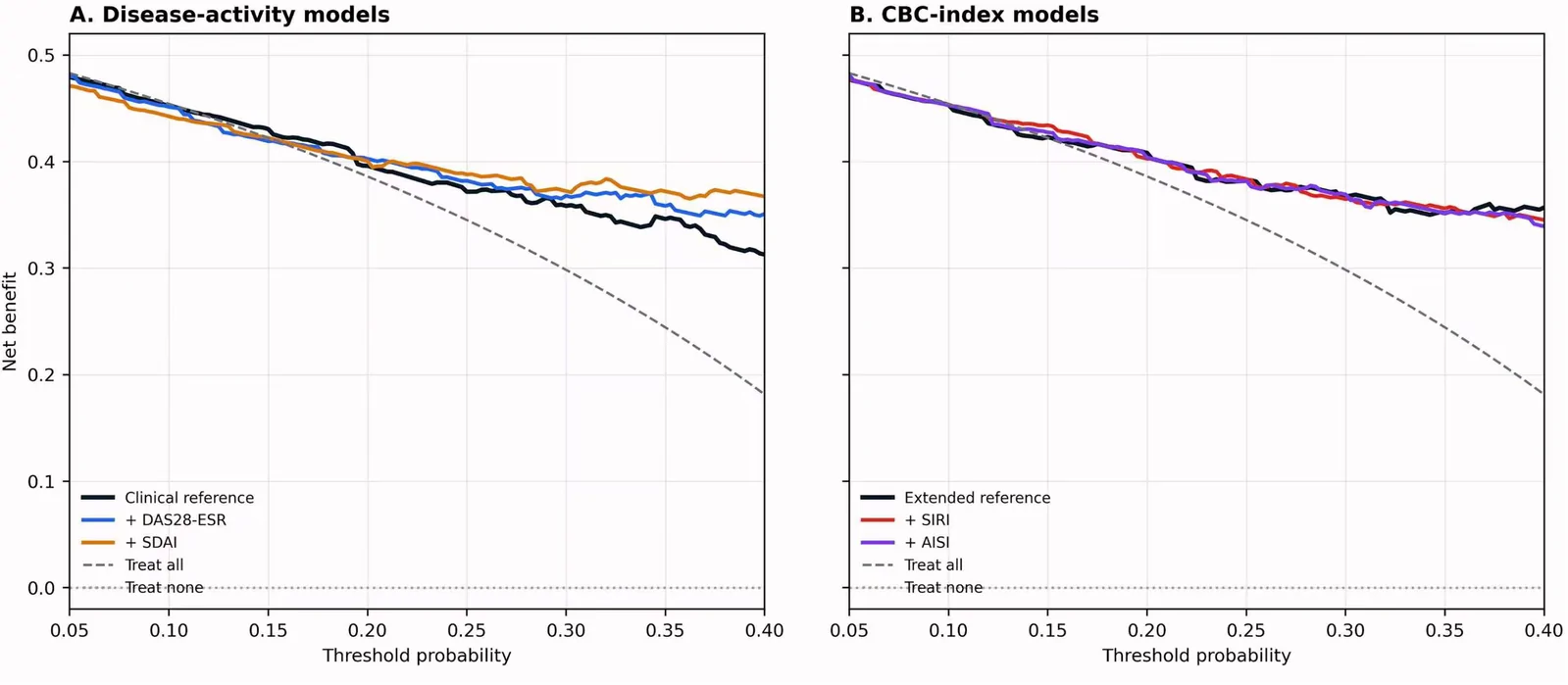
Decision curves for the displayed clinical reference, disease-activity **(A)**, and CBC-derived-index models **(B)**. Disease-activity models showed marginally higher net benefit over portions of the evaluated threshold range, but the curves overlapped substantially and the differences were threshold dependent.

Clinical utility was explored using DCA, with net benefit evaluated across the risk-threshold range displayed in **Figure 1**. Incremental performance was summarized by ΔAUC and exploratory reclassification measures, including continuous NRI and IDI. NRI and IDI were interpreted as model-level reclassification indices rather than as percentages of patients correctly classified.

Restricted cubic splines with four knots placed at the 5th, 35th, 65th, and 95th percentiles of each predictor distribution were used to evaluate the shape of associations between continuous predictors and OP. All spline models were adjusted for age, sex, and BMI. Nonlinearity was assessed by likelihood-ratio tests comparing each spline model (one linear and two nonlinear exposure terms) with the corresponding model containing a single linear exposure term. SIRI and AISI were natural-log transformed for the spline analyses. Curves were plotted over the 2.5th–97.5th percentile range and show marginal predicted probabilities standardized over the observed joint distribution of age, sex, and BMI; pointwise 95% confidence intervals were obtained with the delta method. Nonlinearity P values were interpreted as tests of departure from a linear functional form and not as tests of the overall association. Complete-case data were used. All tests were two-sided, with P<0.05 denoting statistical significance.

### Ethical considerations

The study was conducted in accordance with the ethical principles of the Declaration of Helsinki. The protocol was reviewed and approved by the Ethics Committee of Weifang People’s Hospital, Weifang, China (KYLL20251029-2). Given the retrospective nature of the study and the use of anonymized clinical data, the requirement for written informed consent was waived. All patient data were de-identified before analysis.

## Results

### Baseline characteristics

A total of 226 patients with RA were included, of whom 115 (50.9%) met the criteria for OP. The cohort was predominantly female (91.2%). Patients with OP were older than those without OP (64.7 ± 10.5 vs 57.1 ± 9.1 years, P<0.001) and had longer RA disease duration (60.5 ± 76.5 vs 34.7 ± 26.6 months, P = 0.003) (**Table 3**). They also had a lower BMI (22.9 ± 3.2 vs 25.4 ± 3.5 kg/m², P<0.001). Among women, menopausal status was more frequent in the OP group. Consistent with the outcome definition, the OP group had lower BMD and T-scores and higher FRAX 10-year fracture probabilities. Metabolic or cardiovascular comorbidity, current glucocorticoid use, and anti-osteoporosis treatment were also more frequent in the OP group (**Table 3**).

**Table 3:** Baseline characteristics of RA patients stratified by osteoporosis status.

Variable	Total	Non-osteoporosis	Osteoporosis	SMD	OR (95% CI)	P
Demographics and clinical characteristics						
Sex (female)	206 (91.2%)	98 (88.3%)	108 (93.9%)	0.20	2.05 (0.78–5.34)	0.143
Age, years	60.99 ± 10.50	57.14 ± 9.07	64.71 ± 10.48	0.77	1.08 (1.05–1.12)	<0.001
Disease duration, months	47.82 ± 58.99	34.67 ± 26.62	60.51 ± 76.50	0.45	1.01 (1.00–1.02)	0.002
Menopausal status	181 (80.1%)	79 (71.2%)	102 (88.7%)	0.45	3.18 (1.57–6.45)	0.001
BMI, kg/m²	24.11 ± 3.57	25.36 ± 3.50	22.90 ± 3.20	0.73	0.80 (0.73–0.88)	<0.001
≥1 metabolic/cardiovascular comorbidity	22 (9.7%)	3 (2.7%)	19 (16.5%)	0.48	7.12 (2.04–24.83)	0.002
RF/anti-CCP double negative	56 (24.8%)	34 (30.6%)	22 (19.1%)	0.27	0.54 (0.29–0.99)	0.047
RA activity and routine inflammation						
ESR, mm/h	29.43 ± 24.71	23.38 ± 20.84	35.27 ± 26.75	0.50	1.02 (1.01–1.04)	<0.001
CRP, mg/L	10.52 ± 15.79	9.68 ± 15.07	11.33 ± 16.49	0.10	1.01 (0.99–1.02)	0.435
DAS28-ESR	3.56 ± 1.12	3.07 ± 0.91	4.02 ± 1.11	0.94	2.54 (1.85–3.47)	<0.001
DAS28-CRP, recalculated	3.04 ± 0.95	2.69 ± 0.83	3.38 ± 0.93	0.78	2.43 (1.73–3.41)	<0.001
CDAI, recalculated	8.99 ± 7.03	6.14 ± 5.24	11.75 ± 7.44	0.87	1.15 (1.09–1.21)	<0.001
SDAI, recalculated	10.05 ± 7.53	7.11 ± 5.82	12.88 ± 7.92	0.83	1.13 (1.08–1.19)	<0.001
CBC-derived indices						
NLR	2.84 ± 2.02	2.66 ± 1.93	3.01 ± 2.09	0.17	1.10 (0.95–1.27)	0.207
PLR	174.81 ± 81.84	170.97 ± 83.80	178.53 ± 80.09	0.09	1.00 (1.00–1.00)	0.489
MLR	0.30 ± 0.18	0.28 ± 0.17	0.32 ± 0.18	0.24	4.34 (0.84–22.41)	0.080
SII	714.03 ± 540.80	668.26 ± 548.28	758.20 ± 532.13	0.17	1.00 (1.00–1.00)	0.221
SIRI	1.25 ± 0.98	1.11 ± 0.93	1.39 ± 1.02	0.29	1.37 (1.02–1.84)	0.034
AISI	327.08 ± 302.18	285.71 ± 275.56	367.01 ± 321.98	0.27	1.00 (1.00–1.00)	0.049
Bone health and FRAX (exploratory)						
T-score	-2.46 ± 1.76	-1.06 ± 1.26	-3.81 ± 0.91	2.51	Not estimated (outcome-defining)	<0.001
Z-score	-1.21 ± 1.58	-0.09 ± 1.30	-2.29 ± 0.96	1.94	0.07 (0.03–0.14)	<0.001
BMD, g/cm²	0.52 ± 0.17	0.65 ± 0.13	0.39 ± 0.09	2.40	Not estimated (outcome-defining)	<0.001
10-year probability of major osteoporotic fracture, %	6.07 ± 3.87	4.46 ± 2.66	7.62 ± 4.22	0.89	1.32 (1.19–1.45)	<0.001
10-year probability of major osteoporotic fracture ≥10%	38 (16.8%)	7 (6.3%)	31 (27.0%)	0.58	5.48 (2.30–13.08)	<0.001
10-year probability of hip fracture, %	2.44 ± 2.82	1.28 ± 1.56	3.57 ± 3.28	0.89	1.57 (1.33–1.86)	<0.001
10-year probability of hip fracture ≥3%	63 (27.9%)	11 (9.9%)	52 (45.2%)	0.86	7.50 (3.64–15.46)	<0.001
History of fracture	12 (5.3%)	3 (2.7%)	9 (7.8%)	0.23	3.06 (0.81–11.60)	0.101
Medications						
Current glucocorticoid use	75 (33.2%)	28 (25.2%)	47 (40.9%)	0.34	2.05 (1.16–3.61)	0.013
Basic anti-osteoporosis therapy	161 (71.2%)	67 (60.4%)	94 (81.7%)	0.49	2.94 (1.60–5.39)	<0.001
Bisphosphonate/other antiresorptive therapy	31 (13.7%)	2 (1.8%)	29 (25.2%)	0.73	18.38 (4.27–79.18)	<0.001

Values are n (%) or mean ± SD unless otherwise indicated. AISI, aggregate index of systemic inflammation; BMD, bone mineral density; BMI, body mass index; CDAI, Clinical Disease Activity Index; CI, confidence interval; CRP, C-reactive protein; DAS28, Disease Activity Score in 28 joints; ESR, erythrocyte sedimentation rate; MLR, monocyte-to-lymphocyte ratio; NLR, neutrophil-to-lymphocyte ratio; OR, odds ratio; PLR, platelet-to-lymphocyte ratio; SDAI, Simplified Disease Activity Index; SII, systemic immune-inflammation index; SIRI, systemic inflammation response index; SMD, standardized mean difference.

Disease-activity measures were higher in the OP group. Mean DAS28-ESR was 4.02 ± 1.11 in patients with OP and 3.07 ± 0.91 in those without OP (P<0.001). Recalculated SDAI was 12.88 ± 7.92 versus 7.11 ± 5.82, recalculated DAS28-CRP was 3.38 ± 0.93 versus 2.69 ± 0.83, and recalculated CDAI was 11.75 ± 7.44 versus 6.14 ± 5.24 (all P<0.001). The corresponding standardized mean differences ranged from 0.78 to 0.94. ESR was higher in the OP group, whereas CRP did not differ significantly (11.33 ± 16.49 vs 9.68 ± 15.07 mg/L, P = 0.435).

Among CBC-derived indices, SIRI was higher in the OP group (1.39 ± 1.02 vs 1.11 ± 0.93, P = 0.034), and AISI showed a small between-group difference (367.01 ± 321.98 vs 285.71 ± 275.56, P = 0.049). The other indices showed no statistically significant unadjusted differences (**Table 3**).

### Associations from multivariable regression

The model sequence evaluated the effect of adding disease activity and then a data-informed, exploratory CBC-derived index to a clinically selected adjustment set; it was not an exhaustive search for an optimal variable combination. Model 1 included age, sex, BMI, current glucocorticoid use, and RF/anti-CCP seropositivity; Model 2 added CDAI; and Model 3 added SIRI. Parallel models evaluated all six CBC-derived indices on the same basis.

In the multivariable logistic regression analysis, older age, female sex, and lower BMI were independently associated with OP (**Table 1**). In Model 1, each 1-SD increase in age was associated with an OR of 2.85 (95% CI, 1.96–4.15; P<0.001), female sex with an OR of 5.33 (95% CI, 1.72–16.51; P = 0.004), and each 1-SD increase in BMI with an OR of 0.39 (95% CI, 0.27–0.56; P<0.001). RF/anti-CCP seropositivity was associated with OP in Model 1 (OR, 2.31; 95% CI, 1.10–4.87; P = 0.027), although this association attenuated in the subsequent models.

Models 2 and 3 converged and contained 19.2 and 16.4 OP events per predictor parameter, respectively. Current glucocorticoid use and standardized CDAI were strongly correlated (r=0.839); their VIFs were 3.95 and 3.94 in Model 2 and 4.01 and 3.94 in Model 3. The glucocorticoid coefficient reversed direction after CDAI was added, indicating a suppression pattern and conditional coefficient instability. It should not be interpreted as evidence that current glucocorticoid use protects against OP.

After recalculated CDAI was added in Model 2, each 1-SD (7.03-point) higher CDAI was associated with an OR of 10.17 (95% CI, 3.99–25.93; P<0.001). In Model 3, CDAI remained associated with OP (OR, 10.77; 95% CI, 4.21–27.55; P<0.001), whereas SIRI was not independently associated (OR, 1.34; 95% CI, 0.90–2.00; P = 0.153). In a sensitivity model omitting current glucocorticoid use, the CDAI association remained positive but was smaller (OR, 2.21; 95% CI, 1.46–3.33; P<0.001), supporting stability of direction but not magnitude (**Supplementary Table 2**). These adjusted cross-sectional associations should not be interpreted as risk ratios or causal effects.

Across the six parallel fully adjusted models, the ORs per 1-SD increase in the CBC-derived indices ranged from 1.08 to 1.34 (all P≥0.142), with apparent AUCs of 0.892–0.895. Thus, no index showed a robust independent association, and the conclusion did not depend on selecting SIRI (**Supplementary Table 1**).

In the multivariable linear regression for hip T-score, age and female sex were inversely associated with T-score, whereas BMI was positively associated (**Table 2**). In Model 2, each 1-SD increase in CDAI corresponded to a 0.94-unit lower hip T-score (95% CI, −1.30 to −0.59; P<0.001). In Model 3, the CDAI coefficient remained similar (β, −0.95; 95% CI, −1.30 to −0.59; P<0.001), whereas the SIRI coefficient was small and nonsignificant (β, −0.14; 95% CI, −0.33 to 0.05; P = 0.137). Adjusted R² increased from 0.352 in Model 1 to 0.421 in Model 2 and 0.424 in Model 3.

### Model discrimination and ROC curve performance

**Figure 2:**
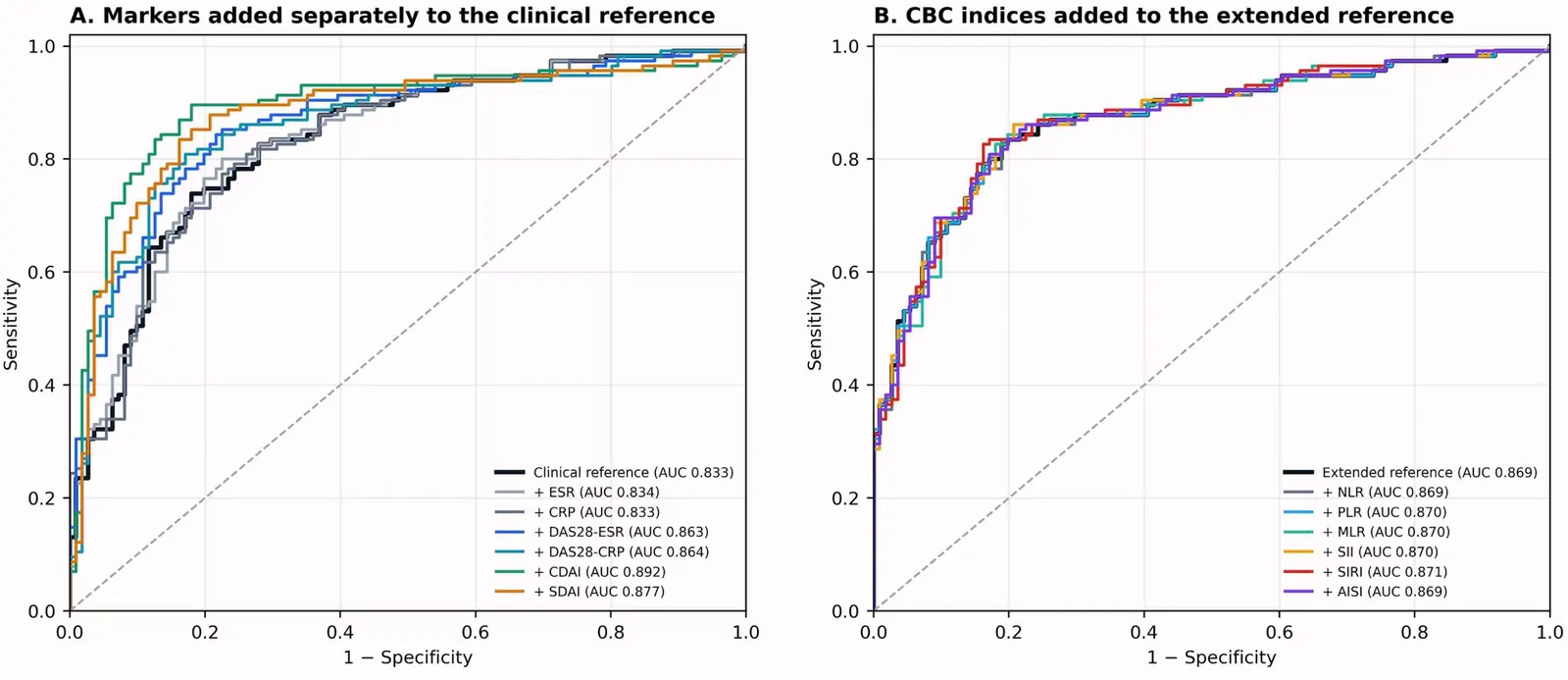
Receiver-operating-characteristic curves comparing the clinical reference model with models additionally including conventional inflammatory markers or disease-activity scores **(A)**, and comparing the extended clinical–disease activity model with models adding CBC-derived indices **(B)**. The curves overlap substantially; complete AUCs and paired comparisons are reported in **Table 4**.

The five-covariate clinical reference model containing age, sex, BMI, glucocorticoid use, and RF/anti-CCP seropositivity achieved an AUC of 0.833 (95% CI, 0.780–0.886) (**Table 4**, **Figure 2**). Adding ESR changed the AUC to 0.834 (ΔAUC, 0.001; P = 0.721), whereas adding CRP alone produced essentially no change (AUC, 0.833; ΔAUC, 0.000; P = 0.958).

**Table 4:** Incremental discrimination of inflammatory markers for prevalent osteoporosis.

Model	AUC (95% CI)	ΔAUC	NRI	IDI	Paired DeLong P
Panel A					
Clinical reference	0.833 (0.780–0.886)	—	—	—	—
+ ESR	0.834 (0.781–0.887)	+0.001 (-0.006 to +0.009)	0.200	0.004	0.721
+ CRP	0.833 (0.780–0.886)	0.000 (-0.006 to +0.006)	0.129	0.001	0.958
+ DAS28-ESR	0.863 (0.814–0.912)	+0.030 (-0.001 to +0.061)	0.637	0.069	0.055
+ DAS28-CRP	0.864 (0.814–0.913)	+0.031 (-0.000 to +0.062)	0.586	0.068	0.052
+ CDAI	0.892 (0.846–0.939)	+0.059 (+0.018 to +0.101)	0.885	0.132	0.005
+ SDAI	0.877 (0.828–0.925)	+0.044 (+0.007 to +0.080)	0.743	0.091	0.018
Panel B					
Clinical reference + DAS28-ESR	0.863 (0.814–0.912)	—	—	—	—
+ CRP	0.869 (0.822–0.917)	+0.006 (-0.011 to +0.023)	0.523	0.019	0.491
Panel C					
Extended reference: clinical reference + DAS28-ESR + CRP	0.869 (0.822–0.917)	—	—	—	—
+ NLR	0.869 (0.822–0.917)	0.000 (-0.004 to +0.005)	0.168	0.003	0.894
+ PLR	0.870 (0.822–0.917)	0.000 (-0.001 to +0.002)	-0.153	0.000	0.467
+ MLR	0.870 (0.822–0.917)	+0.001 (-0.008 to +0.009)	0.239	0.005	0.883
+ SII	0.870 (0.823–0.917)	+0.001 (-0.002 to +0.004)	0.166	0.001	0.545
+ SIRI	0.871 (0.823–0.918)	+0.001 (-0.006 to +0.009)	0.292	0.004	0.724
+ AISI	0.869 (0.822–0.916)	0.000 (-0.005 to +0.005)	0.028	0.000	0.974

The clinical reference model contains age, sex, BMI, glucocorticoid use, and RF/anti-CCP seropositivity. The extended clinical–disease activity model adds DAS28-ESR and CRP. The direct nested comparison without and with CRP is shown. AUC, area under the receiver-operating-characteristic curve; BMI, body mass index; CI, confidence interval; CRP, C-reactive protein; DAS28-ESR, Disease Activity Score in 28 joints using erythrocyte sedimentation rate; IDI, integrated discrimination improvement; NRI, net reclassification improvement.

Adding individual disease-activity scores produced larger, although still modest, absolute changes. DAS28-ESR increased the AUC to 0.863 (ΔAUC, 0.030; P = 0.055), and corrected DAS28-CRP increased it to 0.864 (ΔAUC, 0.031; P = 0.052). CDAI produced the largest observed increase, to an AUC of 0.892 (95% CI, 0.846–0.939; ΔAUC, 0.059; P = 0.005). SDAI increased the AUC to 0.877 (95% CI, 0.828–0.925; ΔAUC, 0.044; P = 0.018). Although the CDAI and SDAI comparisons were statistically significant, the absolute differences remained modest and the ROC curves overlapped substantially; statistical significance alone does not establish clinical importance.

The exploratory reclassification values in **Table 4** were interpreted cautiously. The continuous NRI and IDI were 0.637 and 0.069 for DAS28-ESR and 0.743 and 0.091 for SDAI, respectively. These values were not used to select the extended reference model and should not be interpreted as the proportions of patients correctly reclassified.

The extended clinical/disease-activity reference model containing the five clinical covariates, CRP, and DAS28-ESR had an AUC of 0.869 (95% CI, 0.822–0.917). Adding NLR, PLR, MLR, SII, SIRI, or AISI changed the AUC by −0.0001 to 0.0014 (final AUC, 0.869–0.871; all paired P≥0.467). The corresponding NRI and IDI values were exploratory and were not used to claim clinical improvement. Thus, the CBC-derived indices provided little additional discrimination after conventional clinical factors, CRP, and DAS28-ESR were already included.

In the targeted nested comparison, adding CRP to the five-covariate clinical reference plus DAS28-ESR changed the AUC from 0.863 to 0.869 (ΔAUC, 0.006; 95% CI, −0.011 to 0.023; paired DeLong P = 0.491). This comparison directly quantifies the small incremental discrimination attributable to CRP after clinical factors and DAS28-ESR.

### Clinical utility: decision curve analysis

The decision curves were closely aligned over much of the displayed threshold range (**Figure 1**). Models containing DAS28-ESR or SDAI showed marginally higher net benefit than the clinical reference over selected thresholds, but the curves did not show consistent separation. In the second panel, adding the displayed CBC-derived indices SIRI or AISI produced little separation from the extended clinical/disease-activity reference model. The small and threshold-dependent differences do not establish a clinically important advantage or support a new intervention threshold.

### Spline-based modeling of inflammation and disease activity

**Figure 3:**
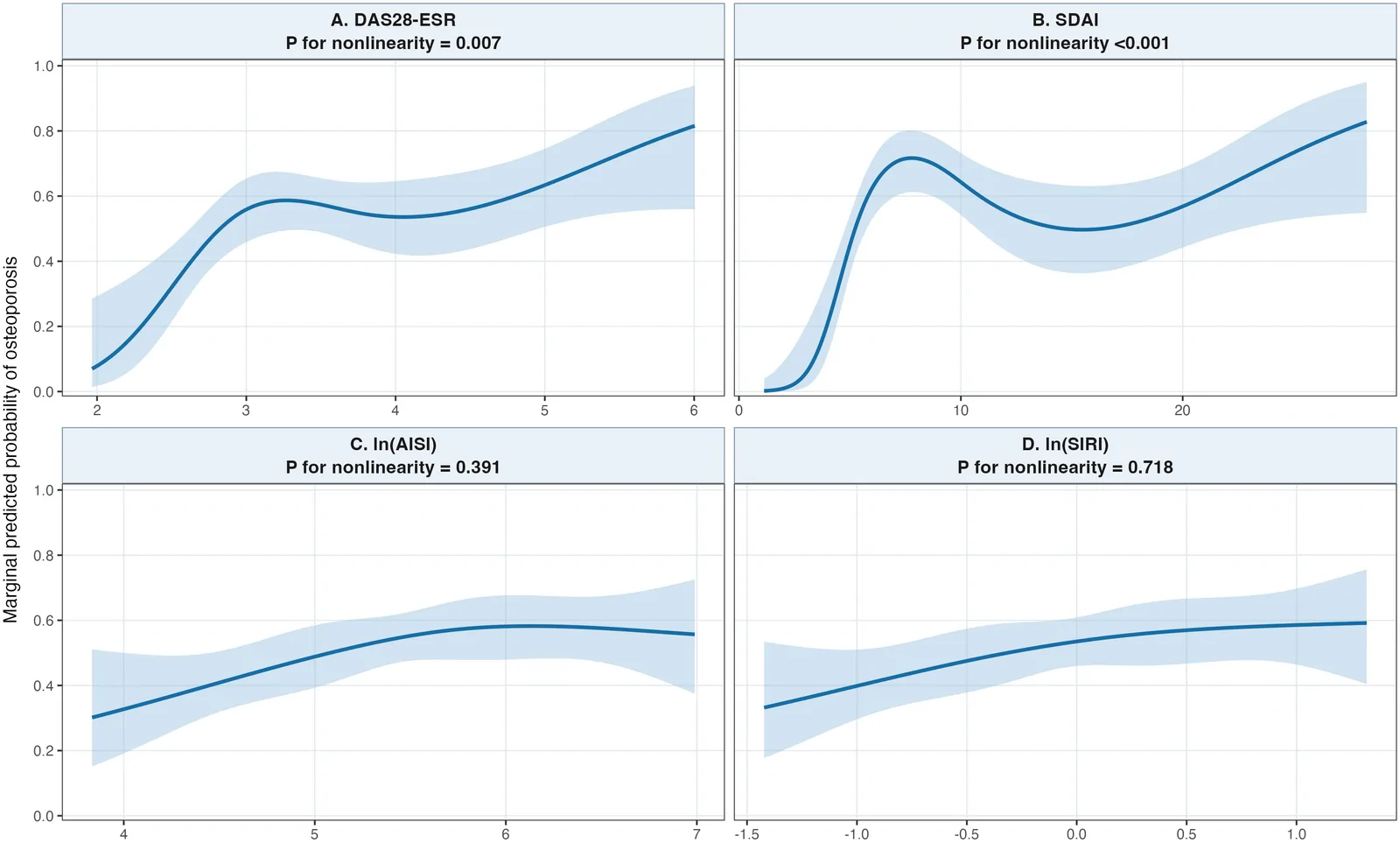
Four-knot restricted cubic spline curves for DAS28-ESR **(A)**, SDAI **(B)**, natural-log-transformed AISI **(C)**, and natural-log-transformed SIRI **(D)**, in displayed panel order. Knots were placed at the 5th, 35th, 65th, and 95th percentiles of each predictor; curves are displayed from the 2.5th to the 97.5th percentile. Models were adjusted for age, sex, and BMI. Curves show marginal predicted probabilities standardized over the observed joint distribution of these covariates, with pointwise 95% confidence intervals obtained using the delta method. Displayed P values are likelihood-ratio tests for nonlinearity comparing the spline model with the corresponding linear-term model. Absence of nonlinearity does not imply absence of an overall association.

Restricted cubic splines were used to examine the functional form of the associations between continuous markers and OP (**Figure 3**). There was evidence of nonlinearity for DAS28-ESR (likelihood-ratio χ²=9.99, 2 df; P for nonlinearity=0.007) and SDAI (χ²=33.49, 2 df; P for nonlinearity<0.001). For these two scores, the marginally standardized curves demonstrated non-monotonic associations across the observed distributions, with pointwise confidence intervals widening toward the extremes; tail behavior should therefore be interpreted cautiously.

There was no evidence of nonlinearity for natural-log-transformed AISI (χ²=1.88, 2 df; P for nonlinearity=0.391) or natural-log-transformed SIRI (χ²=0.66, 2 df; P for nonlinearity=0.718). These nonlinearity tests evaluate the shape of an association rather than whether the overall association is statistically significant. The spline results describe cross-sectional patterns and do not establish that one marker is a more important causal contributor to bone loss than another.

## Discussion

In this single-center study of 226 patients with RA, disease-activity scores were associated with prevalent OP after adjustment for clinical covariates. Adding individual disease-activity scores to the five-covariate clinical reference produced modest improvements in discrimination, with substantial overlap among the ROC and decision curves. By contrast, CBC-derived indices provided little additional discrimination after conventional inflammatory and disease-activity information was included, and none was independently associated in parallel fully adjusted models. The main contribution is therefore the limited incremental value of CBC-derived indices rather than evidence that disease activity is a dominant or causal determinant of bone fragility.

These findings are broadly consistent with prior literature on RA, inflammation, and bone loss. RA is associated with important systemic morbidity, including excess mortality (16), as well as increased OP and fracture burden (17). Skeletal risk has also been documented in male patients with RA (18), in studies of hip OP among postmenopausal women (19), and in relation to rheumatoid factor positivity (20). Longitudinal observations have shown that patients with persistently higher time-averaged disease activity experience greater BMD decline, whereas lower sustained activity is associated with relatively preserved BMD (21). The present cross-sectional results are compatible with this literature but cannot determine temporal direction or show that lowering current disease activity will improve subsequent bone outcomes.

CBC-derived ratios have been proposed as convenient markers of systemic inflammation and bone risk. In older adults, higher inflammatory indices have been associated with greater OP severity (22), and NLR and PLR correlate with RA disease activity in pooled analyses (23). In postmenopausal women with RA, NLR, PLR, and MLR have been associated with OP and incident vertebral fracture (6). In the present study, SIRI and AISI showed small unadjusted differences between OP groups, but none of the six CBC-derived indices showed a robust independent association in parallel adjusted models or materially improved AUC after CRP and DAS28-ESR were included. This distinction is important: an unadjusted group difference does not necessarily add clinically meaningful classification information beyond established variables.

The comparison of acute-phase reactants with composite activity scores also warrants cautious interpretation. Generalized bone loss has been observed in early RA cohorts in the modern treatment era (24), while CRP, ESR, and DAS28-CRP each have recognized strengths and limitations as measures of RA activity (25). Previous work has developed multivariable OP risk models in RA (26), emphasizing the need to assess a marker within an explicit clinical reference rather than in isolation. In the current analysis, ESR and CRP alone produced minimal AUC changes, whereas composite scores produced somewhat larger but still modest changes. Time-averaged DAS28 has also been associated with cardiovascular outcomes (27), illustrating that longitudinal composite scores can reflect broader systemic disease burden, but this does not establish their clinical value for OP screening. Exploratory four-knot RCS analyses suggested nonlinear functional forms for DAS28-ESR and SDAI, whereas no departure from linearity was detected for ln(AISI) or ln(SIRI). Given the cross-sectional design, modest sample size, and imprecision at the distributional extremes, these shape findings should not be interpreted causally or as evidence of clinically actionable thresholds.

Studies outside RA have reported associations between inflammatory markers and BMD (28), and MLR has been evaluated as a predictor in patients with OP (29). Such observations support the biological plausibility of cell-count indices as markers of inflammatory burden. However, the current results do not support interpreting these indices as replacements for established clinical assessment. Their limited incremental performance may reflect their nonspecificity and overlap with inflammation already represented by composite RA activity scores and conventional acute-phase reactants.

From a practical perspective, these findings do not support routinely calculating NLR, PLR, MLR, SII, SIRI, or AISI solely to identify prevalent OP when established clinical variables and RA disease-activity measures are available. Clinicians should continue to base OP evaluation in RA on established clinical risk factors, glucocorticoid exposure, fracture-risk assessment such as FRAX, and DXA when indicated. CBC-derived indices should not be used to replace these assessments, withhold DXA, or determine treatment.

FRAX includes RA as a binary fracture-risk factor, and an individual-participant meta-analysis has confirmed that RA contributes to subsequent fracture risk beyond several conventional variables (30). The level of RA activity is not incorporated into the current FRAX input structure. The observed associations raise the hypothesis that disease activity could refine fracture-risk assessment, but the present study evaluated prevalent BMD-defined OP rather than incident fracture and did not test an activity-augmented FRAX model. Mechanistically, cytokine effects on bone, RANKL–RANK–OPG signaling, suppression of bone formation, and inflammatory osteoclast precursors provide plausible links between RA inflammation and skeletal loss (31–36); these pathways were not measured here and should not be inferred from the model comparisons.

Contemporary validation studies support the use of FRAX with BMD in RA populations (37). Other cohorts have documented changes in generalized BMD during early RA (38, 39), and current clinical guidance recognizes glucocorticoid exposure as a major consideration in fracture-risk assessment (40). Observations of systemic bone loss in patients receiving inflammation-directed RA therapy further support the relevance of inflammatory burden (41). Nevertheless, these external findings do not justify initiating anti-osteoporosis treatment, escalating RA therapy, or setting a new DXA threshold on the basis of the present cross-sectional analysis.

This study has several strengths. It evaluated multiple disease-activity scores and CBC-derived indices within the same clinical cohort and used complementary measures of association, discrimination, reclassification, decision-curve net benefit, and functional form. The explicit model hierarchy also distinguishes the unadjusted SIRI signal from its lack of independent association and its limited incremental discrimination after clinical and disease-activity variables were included.

Several limitations should be acknowledged. First, the retrospective and cross-sectional design precludes causal inference and may not capture temporal changes in inflammation and bone density. Second, the single-center cohort was predominantly female. This distribution broadly reflects the epidemiology and clinical population of RA; however, the small number of men limits sex-specific inference and generalizability to male populations. Third, OP was defined using DXA-derived BMD, the current diagnostic reference standard, but BMD does not capture all aspects of bone quality or future fracture susceptibility, and incident fracture outcomes and trabecular bone score were not evaluated. Fourth, glucocorticoid exposure was recorded as current use without detailed cumulative dose or duration. Its strong correlation with CDAI yielded unstable conditional coefficients, so neither the glucocorticoid nor CDAI estimate should be interpreted causally. Finally, the modest sample size, the data-informed selection of SIRI, and the absence of external validation limit conclusions about clinical utility, although parallel analyses of all six CBC indices reduced reliance on the selected index.

## Conclusions

In patients with RA, disease-activity scores provided modest incremental discrimination for prevalent OP beyond established clinical factors. CBC-derived indices added little once conventional inflammatory and disease-activity information was included, and the present data do not support calculating them solely for OP identification. Clinicians should continue to use established clinical risk assessment, FRAX, and DXA when indicated; CBC-derived indices should not replace these approaches. These findings do not establish disease activity as a dominant causal determinant or define a new screening or treatment threshold. Prospective studies with incident fracture outcomes and external validation are needed.

## Data Availability

The original contributions presented in the study are included in the article/**Supplementary Material**. Further inquiries can be directed to the corresponding authors.
