# Supplementary material for: Evaluating disease activity scores and CBC-derived indices in relation to osteoporosis risk in rheumatoid arthritis: a cross-sectional analysis
Source: Front Immunol. 2026 Aug 26;17:1774189. doi: 10.3389/fimmu.2026.1774189 (PMC13553796; doi:10.3389/fimmu.2026.1774189)
Supplement: Supplementary file 1 [file Table1.docx]

**Supplementary Tables**

**Evaluating Disease Activity Scores and CBC-Derived Indices in Relation to Osteoporosis Risk in Rheumatoid Arthritis: A Cross-Sectional Analysis**

Manuscript ID: 1774189

**Supplementary Table S1. Parallel adjusted logistic-regression models for the six CBC-derived indices**

| **CBC-derived index** | **Adjusted OR per 1 SD** | **95% CI** | **P** | **Apparent AUC** |
| --- | --- | --- | --- | --- |
| NLR | 1.18 | 0.83–1.67 | 0.366 | 0.8942 |
| PLR | 1.08 | 0.78–1.49 | 0.657 | 0.8923 |
| MLR | 1.30 | 0.92–1.84 | 0.142 | 0.8940 |
| SII | 1.14 | 0.80–1.62 | 0.466 | 0.8929 |
| SIRI | 1.34 | 0.90–2.00 | 0.153 | 0.8948 |
| AISI | 1.27 | 0.84–1.92 | 0.260 | 0.8941 |

Each CBC-derived index was standardized to 1 sample SD and added separately to the same Model 2 covariate set: age, sex, BMI, current glucocorticoid use, RF/anti-CCP seropositivity, and CDAI. The indices were not entered simultaneously because they share blood-cell components. AISI, aggregate index of systemic inflammation; AUC, area under the receiver-operating-characteristic curve; CDAI, Clinical Disease Activity Index; CI, confidence interval; MLR, monocyte-to-lymphocyte ratio; NLR, neutrophil-to-lymphocyte ratio; OR, odds ratio; PLR, platelet-to-lymphocyte ratio; SII, systemic immune-inflammation index; SIRI, systemic inflammation response index.

**Supplementary Table S2. Model diagnostics and glucocorticoid-omission sensitivity analysis**

| **Diagnostic** | **Model 2** | **Model 3** |
| --- | --- | --- |
| OP events / predictor parameters | 115 / 6 (19.2) | 115 / 7 (16.4) |
| Model converged | Yes | Yes |
| Condition number | 10.76 | 10.37 |
| VIF: current glucocorticoid use | 3.95 | 4.01 |
| VIF: CDAI | 3.94 | 3.94 |
| Apparent AUC | 0.8923 | 0.8948 |

Model 2 included age, sex, BMI, current glucocorticoid use, RF/anti-CCP seropositivity, and CDAI; Model 3 additionally included SIRI. The Pearson correlation between current glucocorticoid use and standardized CDAI was 0.839. The coefficient pattern is consistent with suppression and conditional instability and should not be interpreted causally.

**Sensitivity estimate for CDAI**

| **Analysis** | **CDAI OR per 1 SD** | **95% CI** | **P** |
| --- | --- | --- | --- |
| Primary Model 2 | 10.17 | 3.99–25.93 | <0.001 |
| Model 2 omitting current glucocorticoid use | 2.21 | 1.46–3.33 | <0.001 |

CDAI was standardized to 1 sample SD (7.03 points). The sensitivity analysis supports stability of the positive direction but not the magnitude of the conditional CDAI coefficient.
